# Prescription Department

**Published:** 1892-01

**Authors:** 


LaGrippe—
The following prescriptions are used by Dr. V. W. Gayle, of Kansas City, Mo. Where there is much angina with acute bronchial irritation, the following is indicated:

R. Ammon. chloride dr. ii.
Potassi chlorat dr. i.
Tinct. ferri chloridi f. dr. ii.
Syr. simplex f. oz. ii.
Aquæ q. s. ft. f. oz. iv.

M. Sig. Teaspoonful in sweetened water every four hours, also apply to the throat with mop every three hours.

Quinine is the best germ destroyer we have for the microbe of influenza. During the recent epidemic I aborted quite a number of cases with antikamnia in combination with salol and quinine. The relief obtained by the administration of antikamnia where the cephalalgia was severe as in the majority of my cases, was wonderful. When the pain seemed almost intolerable, I have seen a ten grain dose of antikamnia banish it. The combination spoken of was as follows:

R. Antikamnia dr. i.
Salol - dr. ss.
Quinia sulph. dr. i.
M. ft. capsules No. XXX.

Sig. One every two hours.

Expectorants are often needed, and antikamnia should be administered with them thus:

R. Antikamnia dr. i.
Syr. senega f. oz. i.
Vini ipecac f. dr. iii.
Syr. tolutan q. s. ft. f. oz. iv.
M. Sig. Teaspoonful every two hours.

The mild chloride of mercury in minimum doses often repeated will be beneficial. The following prescription is a favorite of mine:

R. Hydrarg. chlo. mite gr. i.
Sodii bicarb - scr. i.
Lactopeptine - dr. ss.
M. ft. Charts No. x.

Sig. One every hour until all are taken. This to be followed by a full dose of hunyadi janus or rubinat condal water.—*Med. World.*

Syrup for Infantile Constipation—

℞. Podophyllin, gr. j.
Alcohol, dr. iss.
Syrup of red raspberry, oz. iij

M. Dose, from a teaspoonful to a dessertspoonful every morning, according to the obstinacy of the constipation.—*L' Union Medicale.*

Bilious Headache.—

℞. Pulv. opii,

Pulv. ipecac, aa gr. 1-8th

Mass. hydrarg. gr. ss.

M. ft. tab. trit. No. i.—*N. Y. Med. Journal.*

Nocturnal Emissions.—

℞. Ext. aloes, gr. iij.
Ext. belladonna. gr. ij.
Ext hyoscyami, gr. iv.
Pulv. camphoræ gr. xxiv.

M. ft. pilulæ No. xii. Sig., one pill every night.—*Ex.*

Poison Oak Eruption.—

℞. Ex. Goulards' oz. ij.
Oil olivæ, oz. j.

M. External use in poison oak eruption followed by benz. zinc ointment.

An Injection for Gonorrhœa.—

Prof. Keen gave the following formula to be used as an injection in gonorrhœa:

℞. Zinci sulphat., gr. xviij.
Catechu,
Matico, aa f. dr. iss.
Glycerini,
Aquæ, aa q. s. ad. f. oz. vj.

M. Sig. Inject f. oz. ss., retaining each injection for at least five minutes. —*Times and Register.*

Tonic and Laxative.—

℞. Aloes pulvis, dr. j.
Ferri. sub. carb., dr. ss.
Ex. Hyoscyama, dr. ss.
Ex. belladonna, gr. v.
Ex. nux vomica, gr. viij.

M. ft. mass. div. pill vel. caps. No. 30. Sig. One, a. m. and p. m.—*Ex.*

Cardiac Debility.—

℞. Ferri tartarat., gr. v.
Pot. bicarb., gr. v.
Tr. digitalis, m x.
Infus. calumbæ, oz. j.

M. Sig. To be taken three times a day.

